# Genetic Spectrum and Distinct Evolution Patterns of SARS-CoV-2

**DOI:** 10.3389/fmicb.2020.593548

**Published:** 2020-09-25

**Authors:** Sheng Liu, Jikui Shen, Shuyi Fang, Kailing Li, Juli Liu, Lei Yang, Chang-Deng Hu, Jun Wan

**Affiliations:** ^1^Department of Medical and Molecular Genetics, Indiana University School of Medicine, Indianapolis, IN, United States; ^2^Collaborative Core for Cancer Bioinformatics (C^3^B) shared by Indiana University Simon Comprehensive Cancer Center and Purdue University Center for Cancer Research, Indianapolis, IN, United States; ^3^The Wilmer Eye Institute, Johns Hopkins University School of Medicine, Baltimore, MD, United States; ^4^Department of BioHealth Informatics, Indiana University School of Informatics and Computing, Indiana University – Purdue University Indianapolis, Indianapolis, IN, United States; ^5^Department of Pediatrics, Indiana University School of Medicine, Indianapolis, IN, United States; ^6^Department of Medicinal Chemistry and Molecular Pharmacology, Purdue University, West Lafayette, IN, United States; ^7^Purdue University Center for Cancer Research, Purdue University, West Lafayette, IN, United States; ^8^The Center for Computational Biology and Bioinformatics, Indiana University School of Medicine, Indianapolis, IN, United States

**Keywords:** COVID-19, SARS-CoV-2 genome, evolution, genetic variants, clustering, co-infection

## Abstract

Four signature groups of frequently occurred single-nucleotide variants (SNVs) were identified in over twenty-eight thousand high-quality and high-coverage SARS-CoV-2 complete genome sequences, representing different viral strains. Some SNVs predominated but were mutually exclusively presented in patients from different countries and areas. These major SNV signatures exhibited distinguishable evolution patterns over time. A few hundred patients were detected with multiple viral strain-representing mutations simultaneously, which may stand for possible co-infection or potential homogenous recombination of SARS-CoV-2 in environment or within the viral host. Interestingly nucleotide substitutions among SARS-CoV-2 genomes tended to switch between bat RaTG13 coronavirus sequence and Wuhan-Hu-1 genome, indicating the higher genetic instability or tolerance of mutations on those sites or suggesting that major viral strains might exist between Wuhan-Hu-1 and RaTG13 coronavirus.

## Introduction

A novel betacoronavirus SARS-CoV-2 (Viruses CSGotICoTo, 2020) causing human coronavirus disease 2019 (COVID-19) was first reported in Wuhan, Hubei China in December 2019 (Wu et al., 2020a; Zhou et al., 2020; Zhu et al., 2020). The pandemic of SARS-CoV2 has infected more than 12 million people over 180 countries and areas around the world with a death over a half million as of July 9, 2020 (Dong et al., 2020). The most vulnerable group in this COVID-19 pandemic is elderly and those with different underlying medical conditions such as malnourish, hypertension, diabetes, cancer and cardiovascular abnormality (Guzik et al., 2020). Much effort has been devoted by scientists all over the world to understand the features of SARS-CoV2, particularly the viral genome variations. In some cases, viral genomic mutations play a key role in propagation of SARS-COV-2. Viral mutation may alter the viral infectivity and pose an additional challenge for detection by the host cell, and thus it is critical to identify these mutations, especially in the context of vaccine design and drug development. Similar to other viruses, SARS-CoV-2 has been creating random mutations on the genome over time. Only some of mutations were caught and corrected by the virus’s error correction machinery (Kupferschmidt and Cohen, 2020). Analysing these data can potentially monitor the viral transmission routes and identify novel mutations associated with the transmission (Zhang et al., 2020a). For example, Given 103 earlier genome sequence data, at least two clades of SARS-CoV-2 were found to be involved in the global transmission based on T > C mutation on a singleton site at 28144 of the complete genome, which was further termed as S clade (C28144) and L clade (T28144) (Tang et al., 2020). Evolutionary analyses suggested S clade appeared to be more related to coronaviruses in animals. Most recently, three major clusters of SNVs involved in the pandemic were found by comparing 160 SARS-CoV-2 genomes (Sanchez-Pacheco et al., 2020) with RaTG13 (Forster et al., 2020a). Researchers also employed standard phylogenomic approaches and compared consensus sequences representing the dominant virus lineage within each infected host (Forster et al., 2020a; Lai et al., 2020). Such information will be of important value for the development of vaccine, transmission monitoring and ultimately the control of the pandemic. However, most of these studies were based on limited numbers of SARS-CoV-2 genomes collected during early pandemic time, which might lead to debating conclusions (Chookajorn, 2020; Forster et al., 2020b; Kupferschmidt, 2020; Mavian et al., 2020; Rambaut et al., 2020; Sanchez-Pacheco et al., 2020). To date, more than 40,000 SARS-CoV-2 whole genome sequences have been uploaded to the online platform The Global Initiative on Sharing Avian Influenza Data (GISAID) database^1^ (Elbe and Buckland-Merrett, 2017; Shu and McCauley, 2017). With the availability of increased sample size following SARS-CoV-2 spreading to almost all countries/areas in the world, it is feasible to provide a comprehensive and updated analysis of the viral genetic variations.

In this study, we took advantage of the mega-datasets collected by GISAID which published almost thirty thousand high-quality SARS-CoV-2 genomes with high coverage until June 15, 2020. Our comprehensive analyses clearly revealed distinct patterns of four major group mutations prominent in different countries and areas, suggesting representative SARS-CoV-2 strains correspondingly. We uncovered novel dynamic transmission and evolution patterns for groups of SARS-CoV-2 variants. A few hundred patients were found to have multiple groups of mutations simultaneously. Comparing with four bat coronavirus genomes, we found that alternations of nucleotides on SARS-CoV-2 genome tend to occur at the same sites where bat coronavirus sequences were different from Wuhan-Hu-1. Strikingly, some nucleotide substitutions on SARS-CoV-2 were apt to be the same as RaTG13 coronavirus sequences. We further investigated protein structure alternations caused by the amino acid (AA) changes due to high-frequent non-synonymous SNVs. Our novel genome-wide discoveries provided more detailed information and shed the light of studying SARS-CoV-2 which has been clouding over the world.

## Materials and Methods

### Collection of Sequences

Complete high-coverage coronavirus sequences were downloaded from GISAID database as of June 15, 2020. 28,212 coronavirus genomes isolated from humans and four bat Rhinolophus affinis were analyzed, including Bat CoVRaTG13 and RmYN02 from Yunnan Province, China, SL-CoVZC45, SL-CoVZXC21 from Zhejiang Province, China. White spaces within the sequences were removed. We aligned these sequences using minimap2 (Li, 2018) with the reference, the complete genome of Wuhan-Hu-1 (GISAID ID EPI_ISL_402125) by Wu et al. (2020a,b). The variants were annotated by ANNOVAR (Wang et al., 2010) using NCBI Reference Sequence: NC_045512.2.

### Mutation Rate

Mutation rate is an important factor to monitor virus propagation and evolution (Zhao et al., 2004). In this study, we compared all other SARS-CoV-2 genome sequences against Wuhan-Hu-1 genome only. Hence we modified the formula (Zhao et al., 2004) to calculate mutation rate such that,

$$\mu =\frac{\sum_{i=1}^{N-1}\frac{m_{i}}{t_{i}}}{\left(N-1\right)\,L}\times 365$$

where μ is the mutation rate per site per year, *N* is the total genomes collected after Wuhan-Hu-1 with detailed time information, *m*_*i*_ is number of mutations of *i*th genome compared to Wuhan-Hu-1, *t*_*i*_ is the time difference in days when *i*th genome was collected after Wuhan-Hu-1, and *L* is the total length of SARS-CoV-2 genome (*L* = 29903).

### Circos Plot

Circos plot (Krzywinski et al., 2009; Gu et al., 2014) was made given the ratios of genomes with SNV at each genome location of SARS-CoV-2. The concurrence ratio between two SNVs, X and Y, was defined as the ratio of the numbers of samples with both X and Y to the minimal number of samples with either X or Y.

$$\mathrm{Concurrence}\,\mathrm{ratio}\,\left(X,Y\right)=\frac{\left|X\,\cap Y\right|}{\text{min}\,\left(\left|X\right|,\left|Y\right|\right)}$$

The connection lines in the Circos plot represent SNV pairs with high concurrence ratios (larger than 0.9).

### Clustering of SNVs

Two-way clustering was performed to categorize the SNVs and samples with a distance function of one minus concurrence ratio on 54 frequent SNVs and about twenty-eight thousand samples.

### Enrichment Analysis

While comparing SARS-CoV-2 genomic mutation sites and sites where Wuhan-Hu-1 varying from bats’ coronavirus, we used hypergeometric model to calculate the statistical significance of the overlaps.

### Protein Structure Analysis

We used PyMOL (Schrödinger, Inc.) to visualize and analyze protein structure for WT (Wuhan-Hu-1) and mutations. Mutagenesis tools in PyMOL was utilized to detect if a clash was generated upon mutation. Properties of AAs were retrieved from the “Table of standard amino acid abbreviations and properties” on the Wikipedia^2^. The solved structures of Spike, nsp3, nsp5, nsp7 and Pol were downloaded from Protein Data Bank (PDB) (Berman et al., 2003): 6vyb for Spike using electron microscopy (Walls et al., 2020), 6w6y for nsp3 using X-ray diffraction method (Michalska et al., 2020), 6lu7 for nsp5 using X-ray diffraction method (Jin et al., 2020), 6wqd for nsp7 using X-ray diffraction method (Kim et al., 2020), 6m71 for Pol using electron microscopy (Gao et al., 2020). Structures of other proteins, e.g., nsp2, nsp4, nsp6, Hel, ExoN, ORF3a, M, ORF8, and N, were predicted by C-I-Tasser model^3^ (Yang et al., 2015; Huang et al., 2020; Zhang et al., 2020b): QHD43415_2 for nsp2, QHD43415_4 for nsp4, QHD43415_6 for nsp6, QHD43415_12 for Hel, QHD43415_13 for ExoN, QHD43417 for ORF3a, QHD43419 for M, QHD43422 for ORF8, and QHD43423 for N.

## Results

### Genetic Variants of SARS-CoV-2

**FIGURE 1:**
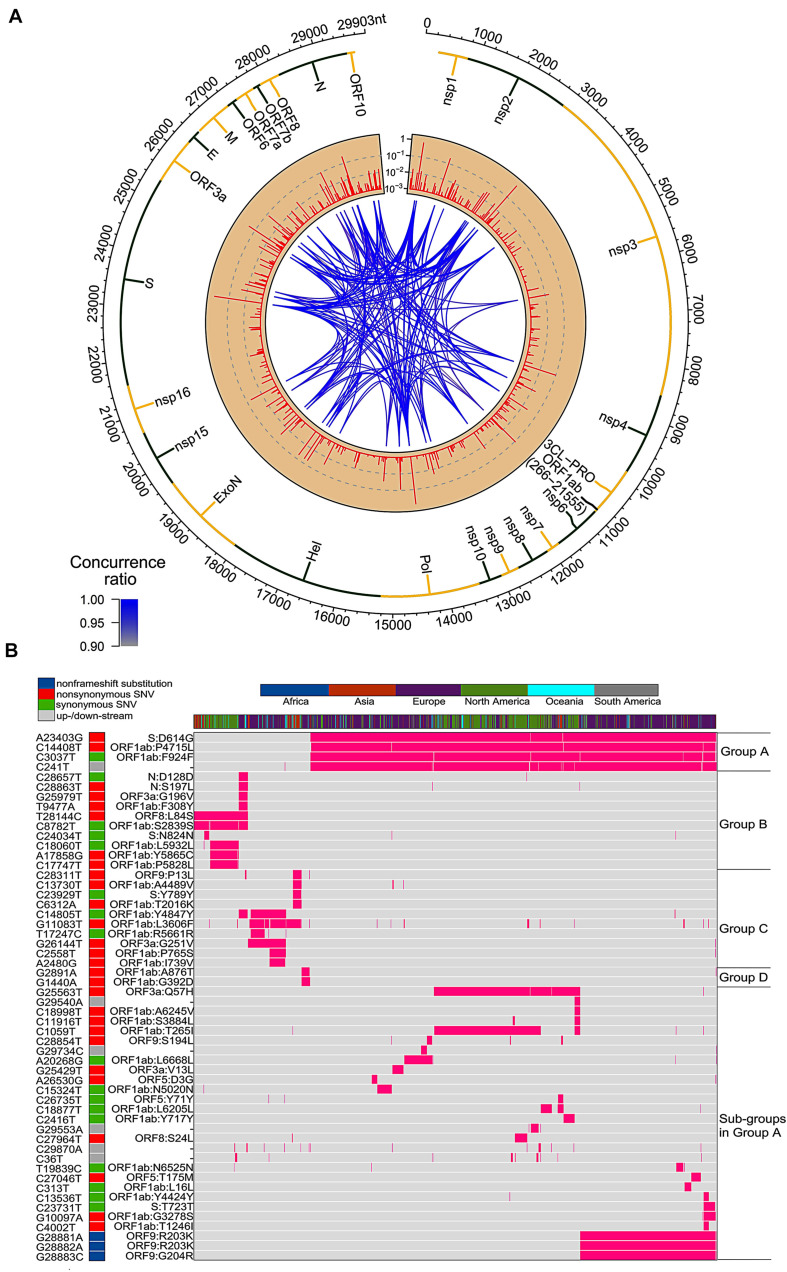
SNVs on about thirty thousand SARS-CoV-2 complete genomes. **(A)** Circos plot shows distribution, frequency, and co-occurrences of SNV2. From outer to inner circle: coronavirus genome location (nt), gene annotation, occurrence ratios of SNVs at the site (log10 scale, red bars), and connections with high concurrence rates (>0.9) represented by blue lines. The darker the blue lines, the higher concurrence rates. **(B)** Fifty-four high frequent SNVs with annotated AA changes were detected (in purple) in about twenty-eight thousand patients worldwide. Four major clusters of SNVs and consequent subgroups can be formed to represent patients from different geographical locations.

We downloaded and analyzed 28,212 SARS-CoV-2 complete genome sequences after excluding low-coverage ones from the GISAID database. Using Wuhan-Hu-1 (NCBI Reference Sequence: NC_045512.2, GISAID ID: EPI_ISL_402125) as reference genome, we found that total 12,649 nucleotide sites had single nucleotide variants (SNVs) when compared to reference genome. The mutation rate was 1.1 × 10^–3^ per site per year for all point mutations, while it became 1.0 × 10^–3^ if we removed SNVs which appeared only once. Both sequence substitution rates were in the same range as 0.80–2.38 × 10^–3^ for SARS-CoV genome as reported (Zhao et al., 2004). Majority of SNVs had very low occurrence frequency (Figure 1A), suggesting a high chance of random or unstable mutations. Four nucleotide substitutions were detected in over 70% of genome sequences: A23403G, C3037T, C14408T, and C241T. They distributed at distinct SARS-CoV-2 genome locations, on the gene body of Spike, ORF1a, and ORF1ab, and upstream of ORF1ab, respectively. Additionally, there were other 50 unique SNVs arose from larger than 1% of populations (*n* > 282). It is interesting that some of these frequent SNVs occurred almost simultaneously with concurrent ratio larger than 0.9 (see methods) as shown by blue-line connections in Figure 1A. They may appear across different proteins. For example, A23403G changes an aspartate to a glycine on Spike (D614G) while C14408T converts a proline to a leucine on ORF1ab (P4715L). Over 99% of both SNVs were found simultaneously on more than 74% samples, suggesting a biological connection between these concurrent variant sites.

Among total 54 frequent SNVs, 31 mutations are non-synonymous variants or non-frameshift substitutions (Figure 1B). Some of them have been discussed separately by previous studies (Forster et al., 2020a; Guan et al., 2020; Tang et al., 2020) or marked as elements in clades G, S, and V from the GISAID report (Elbe and Buckland-Merrett, 2017; Shu and McCauley, 2017). Here, two-way clustering was performed on 54 frequent SNVs and about 28,000 samples (Figure 1B). It is clear to see four major groups of SNVs covering almost all samples, including groups A (C14408T/A23403G, occurring on 21,116 samples), B (T28144C on 2,802 samples), C (G11083T/G26144T on 3,173 samples), and D (G1440A/G2891A on 441 samples). Most SNVs belonged to one unique cluster, while a few SNVs crossed different groups. Taken as an example, a synonymous mutation C14805T existed in both group B and C (Figure 1B), covering over 8% of worldwide samples. Majority (79%) of C14805T can be another signature mutation in group C with SNVs G11083T and G26144T together. In general, the geographical locations of infected patients bearing these special groups of mutations were very different.

**FIGURE 2:**
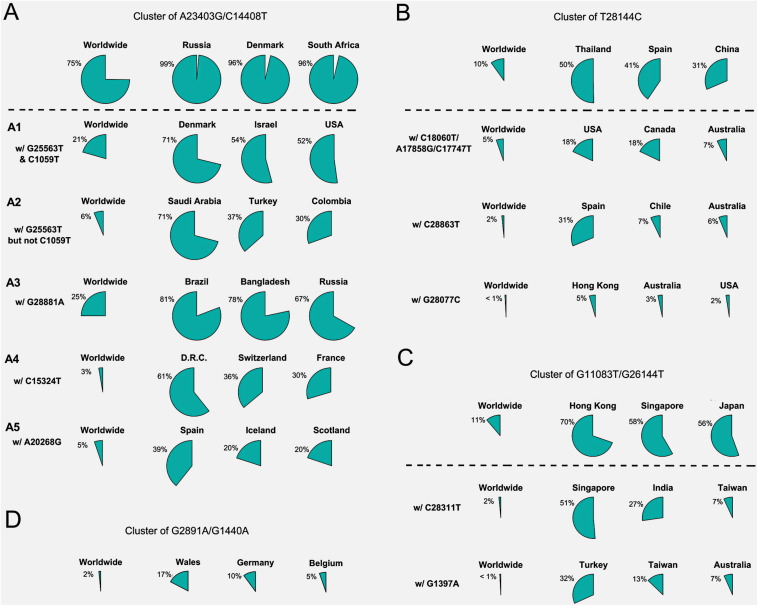
Frequencies of signature SNVs in worldwide and top three countries/areas. **(A–D)** Four major groups and/or their sub-groups had distinct representing countries/areas, indicating different transmission sources and evolution paths.

Forty countries and areas with numbers of viral genomes larger than 50 were chosen to probe the geographical distributions of these SNVs. Group A, represented by two non-synonymous mutations, A23403G and/or C14408T, was borne in totally 72% of samples in the study, including about 82% from Europe and 67% from North America (Figure 1B). The top three countries with the highest ratio in group A (Figure 2A) were Russia (99%), Denmark (96%), and South Africa (96%). Group B was distinguished by non-synonymous mutation T28144C (Figure 1B) which results in substitution of a leucine by a serine on ORF8. It was projected in Thailand (50%), Spain (41%), China (31%), and some other Asian countries/areas (Figure 2B). Group C was featured by two non-synonymous SNVs, G11083T and G26144T (Figure 1B), which substituted a leucine with a phenylalanine on ORF1ab and a glycine with a valine on ORF3a, respectively. This group existed in many Asian and European countries/areas, e.g., Hong Kong, Singapore, Japan, Turkey etc. (Figure 2C), as reported previously (Elbe and Buckland-Merrett, 2017; Shu and McCauley, 2017; Forster et al., 2020a; Guan et al., 2020; Tang et al., 2020). Group D includes two non-synonymous SNVs, G1440A and G2891A (Figure 1B), both of which change the AA sequences on ORF1ab. It confirms the clade D, previously defined by Guan et al. (2020) based on smaller set of patients. G1440A led to the AA change, G212D on non-structural protein 2 (nsp2), while G2891A caused A58T on non-structural protein 3 (nsp3). D-group was mainly found in several European countries/areas, e.g., Wales (17%), Germany (10%), and Belgium (5%) (Figure 2D).

Besides signature variants in each major group discussed above, some SNVs were found in relatively smaller populations but concurred with the major signature SNVs. Importantly, many non-major SNVs were mutually exclusively presented with each other in different countries and areas (Figure 1B). For instance, about 28 mutations coinciding with A23403G and C14408T in the group A composed sub-types of A (Figure 1B), e.g., G25563T and C1059T. However, two separable sets of samples were associated with different combinations of G25563T and C1059T (Figure 2A). Sub-cluster A1 included both G25563T and C1059T, whereas sub-cluster A2 had G25563T but excluded C1059T. They may represent divergent strains found in distinct populations from varied countries/areas. Specifically, A1 occurred in 21% of all SAR-CoV-2 genomes collected world widely, particularly in 71% of Denmark, 54% of Israel, and 52% of United States, whereas A2 was found in only 6% population, which were mostly discovered in Saudi Arabia (71%), Turkey (37%), and Columbia (30%). Another sub-cluster, A3, had consecutive mutations at positions 28881-28883 on SARS-CoV-2 complete genome, leading non-frameshift substitutions on ORF9: R203K-G204R. A3 occupied 25% of worldwide cases, represented by three major countries, Brazil (81%), Bangladesh (78%), and Russia (67%). Even though some sub-clusters of mutations were found in smaller worldwide populations (around or lesser than 5%), they were significantly over-represented in several countries and areas. For instance, A4 with synonymous mutation C15324T was detected in 61% samples of an African country, Democratic Republic of the Congo (DRC), coming together with 36% of Switzerland and 30% of France.

Patients from one country may have different main groups or sub-types of mutations. A synonymous A20268G in cluster A5 (Figure 2A) was sampled in Spain (39%), Iceland (20%), and Scotland (20%). It is interesting that other 41% of Spain samples had another distinguished non-synonymous mutation T28144C in group B (Figure 2B), same as many samples from Asian patients. It suggests the viral transmission path on these patients. 31% of Spain samples also had another unique mutation, C28863T, substituting a serine with a leucine on ORF9, concurrent with T28144C. About 18% of Australia samples were found in group B as well. But they came with additional diverse mutually exclusive SNVs, e.g., either C18060T/A17858G/C17747T (7%), or C28863T (6%), or G28077C (3%) as shown in Figure 2B. Similar scenarios were observed in United States, where approximately 18% of samples encompassed T28144C with C18060T/A17858G/C17747T, while another 2% was recognized with a different non-synonymous mutation G28077C in the same main group B (Figure 2B).

SNVs in group C including G11083T and G26144T existed in many Asian countries and areas (Figure 2C), such as Hong Kong, Singapore, Japan, Indian, Taiwan, and Turkey, as reported previously (Forster et al., 2020a). However, different countries and areas were distinguished by extra variants in the same prime group C. For example, 51% of Singapore was detected with non-synonymous C28311T on ORF9, while Turkey had 32% samples with non-synonymous G1397A on ORF1ab.

**FIGURE 3:**
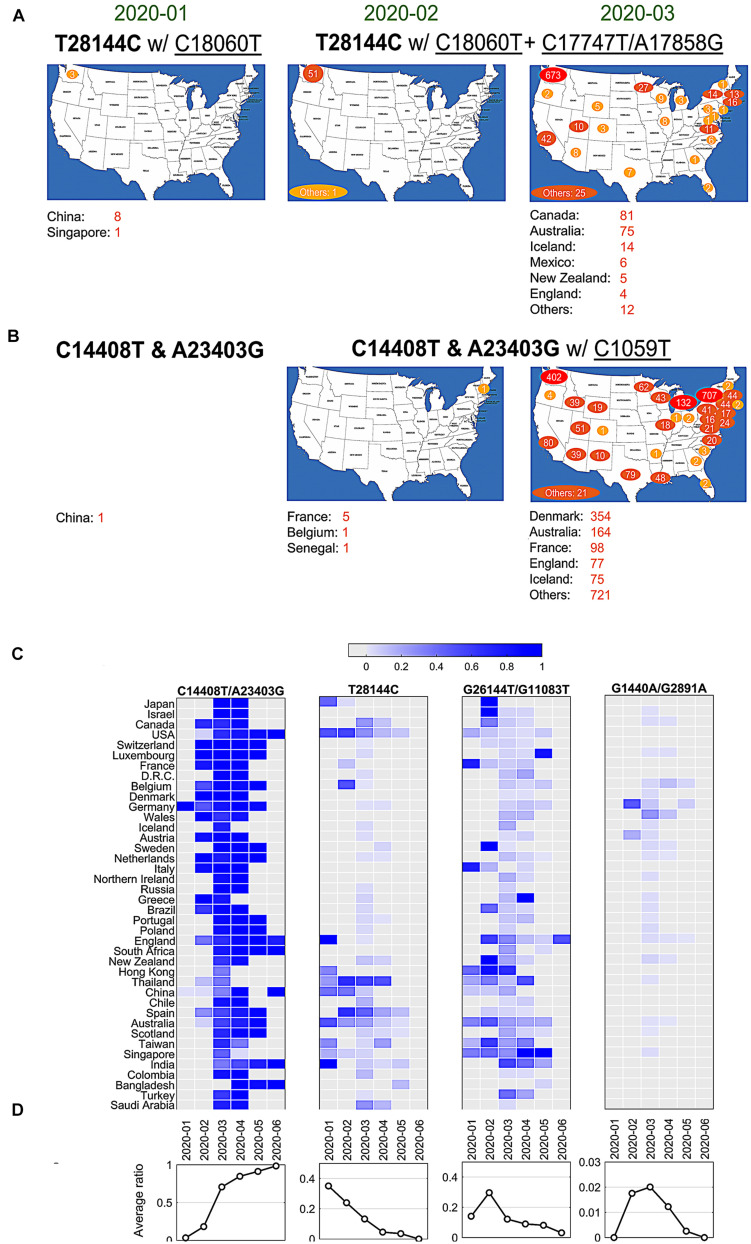
SNVs migration and evolution patterns over time. **(A)** SNV T28144C with C18060T and additional C17747T/A17858G spread in United States and other countries/areas from January to March of 2020. **(B)** SNVs of C14408T and A23403G with C1059T spread in United States and other countries/areas from January to March of 2020. **(C)** The ratios of four significant groups of SNVs, **(A–D)** in Figures 1 and 2, varied in different countries/areas with time development. **(D)** Average temporal ratios of groups **(A–D)** SNVs show distinct patterns from January to June of 2020.

Currently, it lacks sufficient evidences to make a conclusive statement about the origins of all SARS-CoV-2 mutations. But time-annotated data collections can still explore geographical evolution patterning of specific SNVs, albeit limited number of high-quality and high-coverage sequenced viral genomes at some time points. For example, only three cases with mutations T28144C and C18060T (one sub-type in group B) reported in Washington State of United States in January 2020, in addition to eight cases in China and additional one in Singapore at almost the same time (Figure 3A). It is notable that T28144C and C18060T concurred with additional *de novo* non-synonymous mutations C17747T/A17858G on ORF1ab in 51 cases from United States and another one from the cruise of Grand Princess in February 2020. No such case was detected in other countries/areas. One month later, this group of signature variants spread over many states of United States, particularly west coast of United States, and other countries and areas of different continents, including Canada, Australia, Iceland, Mexico, New Zealand, and England etc.

Over half of American patients had been sampled with mutations C14408T/A23403G and C1059T on SARS-CoV-2 genome (Figure 2A). Retrieving data in January 2020, we found only one case with both C14408T and A23403G in China from our dataset (Figure 3B). The first case in United States was reported in New Hampshire at the east coast concurrently with C1059T, in addition to five in France, one in Belgium and one in Senegal. The numbers of such cases boosted up in United States and other countries/areas in March 2020, including 354 in Denmark, 164 in Australia, and 98 in France, 77 in England, etc. In the United States, approximately 1,000 cases were found on the east coast of United States, while over 400 cases were identified on the west coast as well.

The variants of group A (C14408T/A23403G) indicated at least two strains of SARS-CoV-2 distinguishable on the sites of Spike and ORF1ab. One viral strain observed from Wuhan-Hu-1 can be named as DP with an aspartate on 614 of Spike and a proline on 4715 of ORF1ab, while another potential one, named as GL, had a glycine on the site of 614 on Spike and a leucine on the site of 4715 on ORF1ab instead. The ratio of GL strain in all United States cases increased dramatically from 6% in February to 87% in May and June 2020 (Figure 3C). The similar growing trend was observed in most of other countries, regardless when this group of mutations were first present (Figure 3C). In general, 91% of samples from all these countries had strain GL since May 2020 compared to only 3% in February (Figure 3D), suggesting that the GL strain of SARS-CoV-2 might become much more stable and prevailing than the other strain DP like Wuhan-Hu-1 after 6-month evolution and transmission.

Different groups of mutations also exhibited distinguished evolution patterns (Figures 3C,D). Taking B-group SNVs for example, we found that the ratio decreased over time from 35% in January 2020 to almost zero in June in these countries, indicating that at least two strains existed at the early of COVID-19 pandemic. However, strains including variant at 28,144 other than Wuhan-Hu-1 almost diminished after 7 months of transmission. Only the strain that has the same nucleotide T28144 as Wuhan-Hu-1 finally became the most stabilized strain in the host. The similar patterns were observed for groups C and D as well, even though a sudden increasing was found in February and/or March 2020 due to unknown reasons. For instance, in group C with SNVs G1440A and G2891A, Germany had a high ratio, 47.8% (11 out of 23), in February, while 25% of (96 out of 384) Wales were sampled with the same variants in March 2020.

**FIGURE 4:**
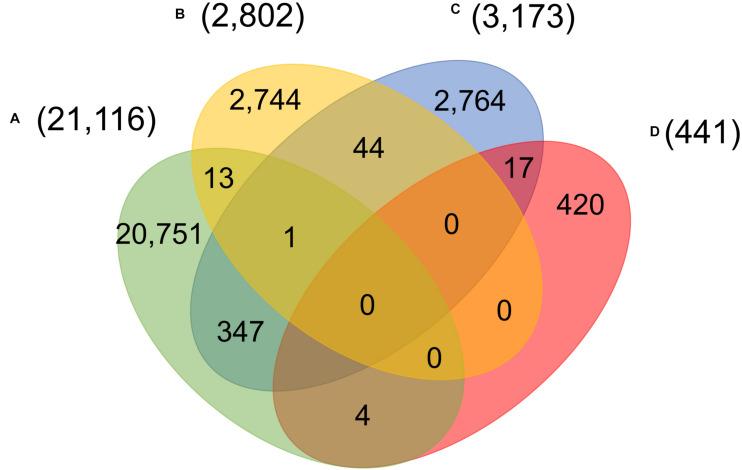
Number of patients carrying significant groups **(A–D)** of SNVs, indicating potential co-infection by different SARS-CoV-2 strains.

Four main groups of mutations showed mutually exclusive in about twenty-eight thousand patients, indicating at least five unique viral strains (including the one same as Wuhan-Hu-1) potentially existed in the host. However, as reported in early of March 2020, a patient hospitalized in Iceland infected by two SARS-CoV-2 subtypes simultaneously^4^. One strain of the SARS-CoV-2 coronavirus was more aggressive, according to Reykjavik Grapevine newspaper citing CEO of CODE Genetics biopharmaceutical company Kari Stefansson. The second strain is a mutation from the original version of the coronavirus that appeared in Wuhan, China. This was regarded as the first known case of co-infection. Gudbjartsson et al. (2020) reported that patient T25 carries both the A2a1a strain and the A2a1a + 25958 strain. As shown in Figure 4, we found that 13 genomes bore both A-group and B-group mutations, while 347 genomes had variant groups of A and C, and both B and C groups were involved in 44 genomes. Strikingly, one patient from Spain was detected with three groups of variants simultaneously, A–C. 17 and 4 out of 441 SARS-CoV-2 genomes with D-group SNVs were overlapped with groups A and C, respectively.

### Comparison of Variants Between SARS-CoV-2 Genomes and Bat Coronavirus Sequences

**FIGURE 5:**
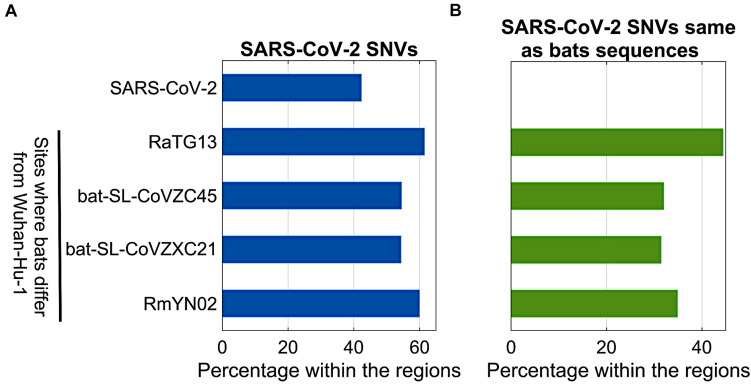
Comparison between SARS-CoV-2 SNVs and nucleotides of bats (RaTG13, bat-SL-CoVZC45, bat-SL-CoVZXC21, and RmYN02) coronavirus varying from Wuhan-Hu-1. **(A)** Percentage of SARS-CoV-2 SNVs on different regions, including SARS-CoV-2 complete genome, and sites where bats coronavirus differ from Wuhan-Hu-1; **(B)** Percentage of SARS-CoV-2 SNVs which converted to bats nucleotides within the same regions as shown in **(A)**.

Bats were regarded as reservoir species for SARS-CoV-2. To understand potential associations between SNVs among SARS-CoV-2 genomes from patients and bat coronavirus sequences, we also aligned four bat coronavirus sequences to Wuhan-Hu-1 complete genome. The ratios of variants between Wuhan-Hu-1 and bats were 3.8% (RaTG13), 11.1% (bat-SL-CoVZC45), 11.1% (bat-SL-CoVZXC21), and 4.8% (RmYN02). As described above, 12,649 out of 29,903 nts (42.3%) on SARS-CoV-2 genome underwent variation among about 28,000 samples. Interestingly, the ratios of SARS-CoV-2 SNVs on the sites where bats’ sequences differed from Wuhan-Hu-1 were significantly elevated (Figure 5A). Among them, RaTG13 reached the highest ratio (61.5%) with *p* = 2.7e-40. The result suggests that the sites where Wuhan-Hu-1 differed from bats might have higher tolerance for sequence variations or higher genetic instability.

In theory, 12,649 identified SARS-CoV-2 SNVs can potentially turn to be any one of three nucleotides other than the original ones from Wuhan-Hu-1. When we focused on the sites where bats coronavirus sequences differed from Wuhan-Hu-1, it turned out that SARS-CoV-2 SNVs had the same mutated nucleotides as RaTG13 coronavirus does on 503 out of 1,132 (44.4%) sites where RaTG13 coronavirus sequence differed from Wuhan-Hu-1 (Figure 5B), including C29095T (Forster et al., 2020a) and seven high frequent SNVs identified from our major groups, e.g., C2416T and C3037T from group A, C8782T, C18060T, C24034T, and T28144C from group B, and C23929T in group C. The ratio for RaTG13 coronavirus was much higher than the ratios observed in other three bat coronavirus sequences (32.0, 31.4, and 34.9%, respectively).

### SARS-CoV-2 SNVs and Protein Functions

Viral sequence mutations will likely affect viral infection, replication, and/or propagation, and thus alter SARS-CoV-2 transmission properties and COVID-19 severity. As previous reported, S68F and P71L non-synonymous mutation in E-protein of SARS CoV-2 were the most common mutation in in E-protein (Hassan et al., 2020); Q57H, G251V, and G196V non-synonymous mutation in ORF3a of SARS-CoV-2 would link to the virulence, infectivity, ion channel activity and viral release (Issa et al., 2020); deletion of ORF8 leads to increased production of the interferon and reduced level of inflammatory cytokines (Chen et al., 2020; Gong et al., 2020; Li et al., 2020a). Most recently, researchers found that D614G non-synonymous mutation located in spike protein would increase infectivity (Eaaswarkhanth et al., 2020; Li et al., 2020b; Yurkovetskiy et al., 2020; Zhang et al., 2020c).

**FIGURE 6:**
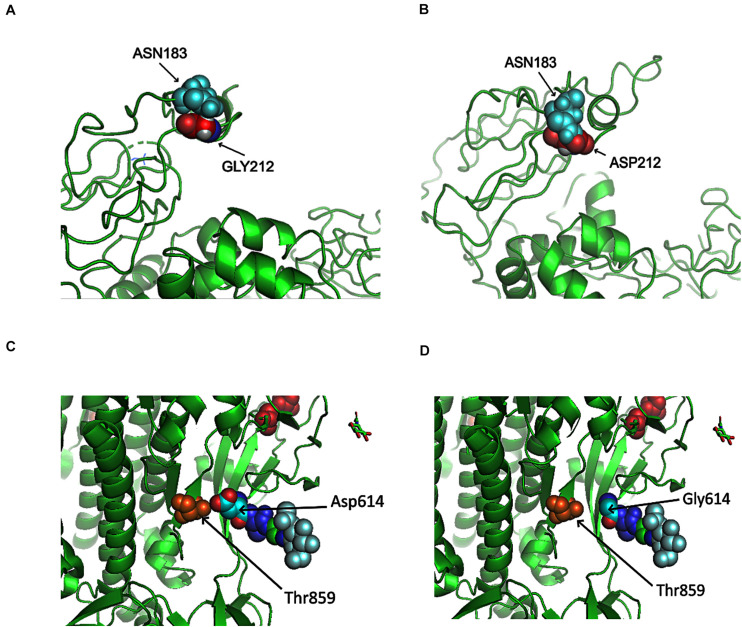
Structures of SARS-CoV-2 non-structural protein 2 (nsp2) and Spike near specific mutation sites. **(A)** No clash was predicted between nsp2 GLY212 and nsp2 ASN183 in Wuhan-Hu-1. **(B)** A clash was predicted between ASP212 and ASN183 after the mutation G212D. **(C)** There was interaction between T859 and D614. **(D)** No contact was precited between T859 and G614 after the mutation D614G.

Here, we analyzed the structure changes of 31 high-frequent non-synonymous SNVs and non-frameshift substitutions (Figure 1B) using PyMOL (Schrödinger, Inc.). It was interesting that all of them were on the surface area of corresponding proteins (Table 1). Eight of them clashed with nearby AAs on non-structural protein 2 (nsp2): G212D, nsp3: A58T, nsp4: F308Y, nsp5: G15S, ORF3a: V13L and G251V, ORF9: G204R, and Pol: A97V, which might be worthy of further analysis. Six of SNVs on S: D614G, ORF3a: Q57H, ORF5: D3G, ORF9: G204R, nsp2: G212D, nsp3: T1198K changed the charge upon the mutations. These changes may contribute to transmission and virulence of SARS-CoV-2. For example, a non-synonymous SNV, G1440A in group D, discovered in over 400 samples from several European countries, led to the AA change of G212D on nsp2. Such a change of G212D may add clashes between residues 212 and ASN183 (Figures 6A,B). It is interesting that Nsp2 G212D falls on the region homologous to the endosome-associated protein similar to the avian infectious bronchitis virus (PDB 3ld1), which plays a key role in the viral pathogenicity (Angeletti et al., 2020).

**TABLE 1:** Protein structures changes corresponding to high frequent non-synonymous SNVs identified.

Protein	SNV	REF Charge	REF Polar	REF Molar mass	ALT Charge	ALT Polar	ALT Molar mass	Side of surface	Predict clash	Size change in molar Mass	Charge change	Polar change	Equivalent protein	Equivalent protein SNV
S	D614G	Negative	Polar	133	Neutral	Non-polar	75	Outer		−57	Yes	Yes		
ORF3a	G251V	Neutral	Non-polar	75	Neutral	Non-polar	117	Outer	Leu219	42	No	No		
ORF3a	G196V	Neutral	Non-polar	75	Neutral	Non-polar	117	Outer		42	No	No		
ORF3a	Q57H	Neutral	Polar	146	Positive	Polar	155	Outer		9	Yes	No		
ORF3a	V13L	Neutral	Non-polar	117	Neutral	Non-polar	131	Outer	Val80	14	No	No		
ORF5/M	D3G	Negative	Polar	133	Neutral	Non-polar	75	Outer		−57	Yes	Yes		
ORF5/M	T175M	Neutral	Polar	119	Neutral	Non-polar	149	Outer		30	No	Yes		
ORF8	S24L	Neutral	Polar	105	Neutral	Non-polar	131	Outer		26	No	Yes		
ORF8	L84S	Neutral	Non-polar	131	Neutral	polar	105	Outer		−26	No	Yes		
ORF9/N	P13L	Neutral	Non-polar	115	Neutral	Non-polar	131	Outer		16	No	No		
ORF9/N	S194L	Neutral	Polar	105	Neutral	Non-polar	131	Outer		26	No	Yes		
ORF9/N	S197L	Neutral	Polar	105	Neutral	Non-polar	131	Outer		26	No	Yes		
ORF9/N	R203K	positive	Polar	174	positive	Polar	146	Outer		−28	No	No		
ORF9/N	G204R	Neutral	Non-polar	75	positive	Polar	174	Inner	Met411	100	Yes	Yes		
ORF1ab	T265I	Neutral	Polar	119	Neutral	Non-polar	131	Outer		12	No	Yes	Nsp2	T85I
ORF1ab	G392D	Neutral	Non-polar	75	Negative	Polar	133	Outer	Asn183	57	Yes	Yes	Nsp2	G212D
ORF1ab	I739V	Neutral	Non-polar	131	Neutral	Non-polar	117	Inner		−14	No	No	Nsp2	I559V
ORF1ab	P765S	Neutral	Non-polar	115	Neutral	Polar	105	Outer		−10	No	Yes	Nsp2	P585S
ORF1ab	A876T	Neutral	Non-polar	89	Neutral	Polar	119	Outer	Ile62	30	No	Yes	Nsp3	A58T
ORF1ab	T1246I	Neutral	Polar	119	Neutral	Non-polar	131	Inner		12	No	Yes	Nsp3	T428I
ORF1ab	T2016K	Neutral	Polar	119	positive	Polar	146	Outer		28	Yes	No	Nsp3	T1198K
ORF1ab	F3071Y	Neutral	Non-polar	165	Neutral	Polar	181	Outer	Phe71	16	No	Yes	Nsp4	F308Y
ORF1ab	G3278S	Neutral	Non-polar	75	Neutral	Polar	105	Outer	Lys97	30	No	Yes	Nsp5	G15S
ORF1ab	L3606F	Neutral	Non-polar	131	Neutral	Non-polar	165	Outer		34	No	No	Nsp6	L37F
ORF1ab	S3884L	Neutral	Polar	105	Neutral	Non-polar	131	Outer		26	No	Yes	Nsp7	S25L
ORF1ab	A4489V	Neutral	Non-polar	89	Neutral	Non-polar	117	Outer	Gln117	28	No	No	Pol/nsp12	A97V
ORF1ab	P4715L	Neutral	Non-polar	115	Neutral	Non-polar	131	Outer		16	No	No	Pol/nsp12	P323L
ORF1ab	P5828L	Neutral	Non-polar	115	Neutral	Non-polar	131	Outer		16	No	No	Hel/nsp13	P504L
ORF1ab	Y5865C	Neutral	Polar	181	Neutral	Polar	121	Outer		−60	No	No	Hel/nsp13	Y541C
ORF1ab	A6245V	Neutral	Non-polar	89	Neutral	Non-polar	117	Outer		28	No	No	ExoN/nsp14	A320V

Similar to SARS coronavirus, SARS-CoV-2 enters into human cells through the binding of its receptor-binding domain (RBD) on Spike protein to human receptor ACE2 (Hoffmann et al., 2020b). Furin is responsible for the proteolytic cleavage of Spike protein to facilitate virus entry. In SARS-CoV-2, 15-nt CCTCGGCGGGCACGT encodes five AAs: PRRAR (681-685), locating at 23603–23617 of Wuhan-Hu-1 complete genome. Furin cleavage site bears a RXXR pattern (Molloy et al., 1992; Shiryaev et al., 2013). R685 makes an ideal furin proteolytic cleavage site (Coutard et al., 2020). Out of almost thirty thousand samples, 58 SARS-CoV-2 genomes were detected with mutations in the region, including 13 from England and 23 from United States. Non-synonymous SNVs, C23604T, was most frequent among others, causing the mutation of P681L. Other AA mutations included P681H/L/S, R682Q/W, R683P/Q, and A684E/T/S/V. As described previously, D614G on Spike caused by the SNV A23403G in group A covered about three-quarter of total sequenced genomes in our study. D614G on Spike did not generate clashes from the protein structure predictions (Figures 6C,D). However, the residue variations changed the negative polar side chain to neutral non-polar side chain (Figures 6C,D and Table 1). Since the site is close to the furin region, such alternation might be able to affect the interactions between furin and furin cleavage sites, then further influence cell-cell fusion and ability to infection (Hoffmann et al., 2020a).

In addition, Cryo-EM-based structural analysis (Wrapp et al., 2020) revealed that 5 key AAs within 434–507 of Spike protein contributed most to the binding activity. This was also confirmed by several recent cryo-EM structural studies (Shang et al., 2020a,b; Wang et al., 2020a; Yuan et al., 2020). The key AAs of SARS-CoV-2 RBD are: L455, G482, V483, E484, G485, F486, Q493, S494, and N501. Interestingly, we identified several non-synonymous SNVs of SARS-CoV-2 on L455, V483, G485, and S494 from sequenced sample, for instance, G22927T (L455F), G23009T (V483F), T23010C (V483A), G23105A (G485S), and T23042C (S494P). Among them, 28 viral genomes had mutation T23010C (V483A), all of which were sampled in United States, including 26 from Washington State. The RBD for SARS-CoV-2 has residues and motifs found in all three clades in lineage B of betacoronavirus (Letko et al., 2020), suggesting distinct cell entry pattern than that of other clades. L455, G485, F486, and N501 are among contact points of virus to human ACE2, changes in these positions may affect the strength of transmission of the virus.

## Discussion

In this study, we comprehensively analyzed almost thirty thousand high-quality and high-coverage SARS-CoV-2 complete genome sequences as well as four bat genome sequences. Even though some SNVs were reported previously and discussed individually, we used bioinformatics approaches to systematically identify four major mutually exclusive groups of SNVs among all samples, suggesting at least five viral strains existing (including one strain same as the reference). These mutations were detected in populations from different geographical locations. The results could provide some insights of possible new functions of SARS-CoV-2 proteins and further bring therapeutic potentials.

Distinct time-course evolution patterns were observed for four major groups of mutations. Some viral strains, e.g., GL with mutations C14408T and A23403G, may gradually replace Wuhan-Hu-1, to become dominant after several month evolutions. Or others may be eliminated naturally with time development, e.g., strains associated with groups B–D mutations (Figures 3C,D). It is hard to explain aberrant emergence of some strains, e.g., the peak time of groups C and D in February and March (Figures 3C,D), particularly due to the lack of enough numbers of high-quality sequenced samples worldwide, including China and other countries/areas, before February 2020. However, with more and more clinical data generated, evolution patterning associated with specific biological functions may be clearly uncovered. For example, several groups recently reported that A23403G mutation in Spike protein might alter the antigenic property and transmission ability due to the change of Spike-ACE2 interaction (Becerra-Flores and Cardozo, 2020; Korber et al., 2020a).

In general, four SNV clusters were mutually exclusively presented. But we still noticed a few hundred patients who were identified to carry multiple groups of SNVs simultaneously. Without clear evidence that homologous recombination in these regions in the intermediate or human host could occur in these viruses, we just defined such overlaps as potential co-infections based on our observations and current knowledge. One possibility is that two or three strains co-existed and prevailed in the population of the same region during the periods when the patients got infected from other people. The patients could be first infected with one strain then another one later, suggesting that primary infection did not yield immunity in time against the subsequent infection from a different strain. Another possibility is that the virus underwent mutations during the transmission to another human due to the special environment of the host, consequently multiple representative mutations were present on the same patient. There are several other scenarios in addition to co-infections, including doubtful sequencing errors or cross sample contaminations. Unfortunately, it lacks of enough information at this moment about the potential post-infection immunity that has important implications for the epidemiologic assessment for the transmission (Kirkcaldy et al., 2020). Of course, the percentage of co-infection cases was less than 1.5% in this study. It might be the consequences of the quarantine and lockdown policy enforced after the spread of COVID-19, while social distancing and wearing face mask are considered effective approaches in reducing the chance of co-infections (Cheng et al., 2020; Eikenberry et al., 2020; Wang et al., 2020b; West et al., 2020). These policies reduced the likelihood that people met patients with different SARS-CoV-2 strains at the same time.

We further compared SNVs among SARS-CoV-2 genomes from human patients to bat coronavirus sequences. It is interesting that SARS-CoV-2 SNVs, particularly those high-frequent mutations, tend to occur at the same sites where bats coronavirus sequences varied from Wuhan-Hu-1, suggesting the high tolerance of these sites for genetic mutations, or potentials of SARS-CoV-2 turning to a wild-type pathogenic phenotype. RaTG13 coronavirus was most similar to SARS-CoV-2 from perspective of sequences, but it held the highest ratio of SARS-CoV-2 variants which converted to the bat’s coronavirus sequences at the same sites. This suggests that some strains of SARS-CoV-2 deviated from Wuhan-Hu-1 might be more similar to bat coronavirus RaTG13 than other bat coronavirus strains presented in this paper. Of course, we don’t have more evidence to show the exact connections between them, but our results may shed the light to search intermediate host and further understand the mechanisms of interspecies transmission in future.

In addition to ORF proteins, four major structure proteins: Spike (S), Envelope (E), Membrane (M), and Nucleocapsid (N), help SARS-CoV2 in assembling and releasing new copies of the virus within human cell. We found that all high-frequent SARS-CoV2 SNVs occurred on the surface of proteins. One of most frequent mutations, D614G, has been detected to be dominant around the world now (Korber et al., 2020b). This SNV caused more infections than other mutations (Li et al., 2020b). Korber et al. (2020a,b) made suggestions from two frameworks of the potential mechanism of being more infectious: on the structure, D614G disconnects the connection between 614 in S1 and 859 in S2, which in turn facilitates the shedding of S1 from viral-membrane-bound S2 or impacts RBD-ACE2 binding by influencing RBD positioning. On the immunological aspect, D614 is within immunodominant linear epitope. Binding of antibody to the epitope may incur conformational change in Spike resulting in nearby enhanced RBD interaction with ACE2. Since furin cleavage sites are essential for SARS-CoV-2 infection of human, in addition to D614G, variants on or nearby the furin cleavage sites may affect virus entry and spread (Hoffmann et al., 2020a).

In summary, we attempted to uncover fundamental genetic patterns of SARS-CoV-2 which may help us understand functional consequences due to the viral genetic instability. Our efforts in exploring the views of SARS-CoV-2 migration and evolution in different geographical locations can be helpful to fight against the pandemic. Our findings may provide useful insights on SARS-CoV-2 replication, pathogenicity, and implications. We look forward to incorporating our results with other studies, e.g., interaction maps between SARS-CoV-2 proteins and human proteins (Gordon et al., 2020), for drug discovery, antibody design or vaccine development in near future.

## Data Availability Statement

The SARS-CoV-2 genome sequences were downloaded from GISAID, which are subject to GISAID’s terms and conditions (https://www.gisaid.org/registration/terms-of-use/).

## Author Contributions

SL, JS, SF, KL, JL, LY, C-DH, and JW performed the research. SL, JS, and JW analyzed the data and wrote the manuscript. All authors reviewed and revised the manuscript.

## Conflict of Interest

The authors declare that the research was conducted in the absence of any commercial or financial relationships that could be construed as a potential conflict of interest.

## Footnotes

1 https://www.gisaid.org/

2 https://en.wikipedia.org/wiki/Amino_acid

3 https://zhanglab.ccmb.med.umich.edu/COVID-19/

4 https://www.mbs.news/a/2020/03/icelandic-man-reportedly-caught-two-coronavirus-subtypes-simultaneously.html
